# Use of Gases to Treat Cochlear Conditions

**DOI:** 10.3389/fncel.2019.00155

**Published:** 2019-04-24

**Authors:** Jay C. Buckey

**Affiliations:** Space Medicine Innovations Laboratory, Center for Hyperbaric Medicine, Department of Medicine, Geisel School of Medicine at Dartmouth, Lebanon, NH, United States

**Keywords:** hyperbaric, oxygen, hydrogen, cochlea, inner ear

## Abstract

Although the cochlear vascular supply (stria vascularis) is designed to block to certain compounds and molecules, it must enable gas exchange to survive. The inner ear capillaries must deliver oxygen and remove carbon dioxide for the cochlea to function. These gases diffuse through tissues across a concentration gradient to reach the desired target. Tight junctions or the endothelial basement membrane do not impede them. Therefore, gases that can diffuse into the inner ear are attractive as therapeutic agents. The two gases most often used in this way are oxygen and hydrogen, although carbon dioxide, ozone, and argon have also been investigated. Typically, oxygen is delivered as hyperbaric oxygen (HBO) (oxygen at pressure higher than atmospheric) to provide increased oxygen levels to the inner ear. This not only relieves hypoxia, but also has anti-inflammatory and other biochemical effects. HBO is used clinically to treat idiopathic sudden sensorineural hearing loss, and both animal and human studies suggest it may also assist recovery after acute acoustic trauma. Laboratory studies suggest hydrogen works as a free radical scavenger and reduces the strong oxidants hydroxyl radicals and peroxynitrite. It also has anti-apoptotic effects. Because of its anti-oxidant and anti-inflammatory effects, it has been studied as a treatment for ototoxicity and shows benefit in an animal model of cisplatinum toxicity. Gas diffusion offers an effective way to provide therapy to the inner ear, particularly since some gases (oxygen, hydrogen, carbon dioxide, ozone, argon) have important therapeutic effects for minimizing cochlear damage.

## Using Gases to Get Potentially Therapeutic Interventions to the Cochlea

Hyperbaric oxygen (HBO) treatment is the most common example of using gas diffusion to deliver a therapeutic agent to the cochlea. HBO is oxygen provided at levels above atmospheric pressure and is dosed in ATA (atmospheres absolute). One hundred percent oxygen given at normal atmospheric pressure is 1 ATA of oxygen. Most hyperbaric treatments are provided within a pressurized chamber with oxygen at 2.0–2.5 ATA. At these pressures, HBO increases oxygen dissolved in plasma dramatically, which elevates the oxygen content of blood reaching tissues. The driving force for this gas uptake is the basic physics of gases. The partial pressure of a gas in a liquid will match the partial pressure of the gas in the atmosphere around it (Henry’s law). The partial pressure of nitrogen in the body, for example, matches that in the atmosphere. Gases dissolve into tissue throughout the body (blood, muscle, fat) based on their solubility and the inner ear is no exception.

The amount of oxygen that can be delivered under pressure is striking. The partial pressure of oxygen in room air is approximately 150 mmHg. The partial pressure of 100% oxygen is 760 mmHg. HBO at 2.0 atmospheres absolute provides an oxygen partial pressure of 1520 mmHg to the lung, or approximately 10 times the partial pressure in room air. Early studies showed that HBO at sufficient pressure provides enough oxygen to meet all the body’s oxygen needs solely from oxygen dissolved in plasma. For example, the classic study by Boerema et al. demonstrated that when essentially all red blood cells were removed from a pig (i.e., the hematocrit was basically zero) the pig could survive without any signs of ischemia when given 100% oxygen at 3.0 ATA (Boerema et al., 1960; Boerema, 1964). Similarly, Lambertsen et al. (1953) showed that oxygen saturations in venous blood of humans exposed to 3.5 ATA was at arterial levels measured with air at normal atmospheric pressure. The high concentration of oxygen in the plasma created by HBO creates a strong gradient for diffusion into hypoxic tissue.

This same diffusion effect occurs with other gases. For example, divers take on large amounts of the gases they breath at depth. When breathing air, the deeper the diver goes (i.e., the higher the pressure) the greater the amount of nitrogen that dissolves in tissues. At these higher pressures, nitrogen gas has a narcotic effect due to the effect of the dissolved nitrogen on nerve transmission. This produces the hazardous “rapture of the deep,” or nitrogen narcosis, characterized by diminished brain function, and an inability to think clearly. When divers ascend they are at risk for decompression sickness as the gas that was dissolved in tissue starts the process of elimination from the body. If the ascent is too fast, bubbles of the gas may form leading to the characteristic symptoms of the “bends” (e.g., pain in joints, embolic stroke). Although decompression sickness usually results from nitrogen, divers using special gas mixes containing hydrogen can also get decompression sickness as the hydrogen which dissolved in tissue during the dive leaves the tissue as the diver ascends and the pressure around the diver is reduced. Individuals don’t need to dive, however, to increase the amount of a particular gas in the body. Just increasing the concentration of a gas in the atmosphere at normal atmospheric pressure (say 2% hydrogen or 20% argon in air) will increase the uptake of that gas into tissue. Also, hydrogen can be delivered to tissues by ingesting or injecting water or saline saturated with hydrogen.

## Oxygen as a Cochlear Therapeutic

The cochlea has a high oxygen requirement and so is sensitive to disruptions of its blood supply (Thalmann et al., 1972). As described above, the hypoxia-relieving effect of HBO is well known and has been considered its primary mechanism of action for many conditions. In the setting of acute ischemia, HBO creates a large oxygen concentration gradient between well-perfused and poorly perfused areas. The high oxygen tension in the inspired air increases the oxygen dissolved in the blood, which in turn increases tissue oxygen levels and promotes oxygen diffusion into the poorly perfused tissue. This immediate hypoxia relieving effect is the rationale behind the use of HBO for conditions associated with acute interruption of the blood supply (e.g., compromised flaps and grafts, crush injuries).

For the cochlea, hypoxia can result from various insults. Acoustic trauma, for example, can lead to reduced cochlear blood flow and inner ear hypoxia (Lamm and Arnold, 1999). Acoustic trauma can disrupt the microcirculation in the cochlea and lead to local ischemia (Shi, 2016). This may be more of an issue in older individuals since there is atrophy of the stria vascularis over time (Shi, 2016) and so disruption of this atrophic stria could lead to more extensive damage. HBO has been shown to be effective in treating acoustic trauma possibly due to relief of cochlear hypoxia (Kuokkanen et al., 1997; Lamm et al., 1998a,b; Lamm and Arnold, 1999; Fakhry et al., 2007; Bayoumy et al., 2019). Another condition where hypoxia may also play an important role is idiopathic sudden sensorineural hearing loss (ISSNHL). As the name implies, the reason for this hearing loss is not known, but it may well result from the sudden loss or compromise of the cochlear circulation. Another possibility is that inflammation in the cochlea may compromise the stria vascularis and lead to hypoxia. The Undersea and Hyperbaric Medical Society lists ISSNL as an approved indication for HBO treatment, and it is often used successfully for this (Stachler et al., 2012; Ajduk et al., 2017; Cho et al., 2018).

One continuing controversy surrounding the use of HBO to relieve hypoxia is whether it might be detrimental by creating oxidative stress in the compromised region. The high levels of oxygen that HBO provides could increase reactive oxygen species (ROS) within the tissue. ROS are thought to be important factors in causing cochlear damage in both acoustic trauma and cisplatinum toxicity (Kamogashira et al., 2015). Since ROS may underlie some of the cochlear damage resulting from acoustic insults, increasing ROS might be harmful. One rodent study suggests this might be true. Cakir et al. (2006) showed that giving HBO shortly after acoustic trauma due to noise led to worse outcomes. This has not been a universal finding, however. In the study by Kuokkanen et al. (2000) rats were exposed to impulse noise and HBO was given daily for 10 days. Hair cell survival was improved in the HBO treatment group compared to controls (Kuokkanen et al., 2000). Recent studies in humans suggest benefit from HBO after acute acoustic trauma (Bayoumy et al., 2019). Table 1 summarizes some of the main results when HBO has been used in acute acoustic trauma. Overall the results have been positive. This issue of ROS damage from HBO is also a consideration for cisplatinum toxicity, since increased ROS are thought to be an important factor in cisplatinum-induced damage. Cobanoglu et al. (2019) recently reported that HBO treatment of rats after receiving cisplatinum led to improved recovery after the insult compared to the untreated control group. Interestingly, in a study of cisplatinum-induced nephrotoxicity, Aydinoz et al. (2007) showed that once-daily HBO provided benefit, while twice-daily HBO seemed to potentiate the damage.

**Table 1:** Summary of results from studies using hyperbaric oxygen in acute acoustic trauma.

Author	Subjects	Intervention	Outcome measure	Results
Kuokkanen et al., 1997	39 rats, 15 treatment, 24 control	2.5 ATA for 90 min daily for 10 days	ABR thresholds	HBO showed less threshold shift than control *p* = 0.07
Lamm and Arnold, 1999	Multiple groups of 16 guinea pigs compared to 22 controls	2.6 ATA for 60 min, 60 min after noise exposure	Cochlear microphonic, compound action potential, ABR	HBO+ prednisolone provided best recovery compared to other therapies tested
Kuokkanen et al., 2000	3 groups of rats. 14 HBO, 24 exposed no HBO, 10 non-exposed controls	2.5 ATA for 90 min daily for 10 days starting 2–3 h after exposure	ABR, histology	Improved thresholds and less hair cell loss in HBO group
Cakir et al., 2006	4 groups of 6 rats	2.4 ATA for 90 min. Group 1 treated 1 h post-exposure, groups 2,3,4 treated 2, 6, 24, 46 h post-exposure	DPOAEs	Worse recovery in animals given HBO 1 h post-exposure. Possible faster recovery in 2 and 6 h groups
Bayoumy et al., 2019	23 noise exposed humans compared to 18 controls. All received oral steroids	Oral steroids plus 2.5 ATA for 90 min daily for 10 sessions starting 4.4 ± 2.7 days after injury	Audiometry	Significantly better improvement in thresholds in HBO group


*ATA, atmospheres absolute; HBO, hyperbaric oxygen; ABR, auditory brainstem response; DPOAE, distortion product otoacoustic emission.*

One possible reason for the different outcomes is that HBO may have competing effects. While the extra oxygen may increase ROS production, HBO may also reduce neutrophil adhesion (Atochin et al., 2000; Miljkovic-Lolic et al., 2003; Francis et al., 2017) which is a key step in the initiation of inflammation. HBO may also downregulate the expression of COX-2, which could also reduce inflammation and potentially improve outcomes (Yin et al., 2002). A growing body of evidence shows that HBO can affect both inflammation and proteases [e.g., matrix metalloproteases (MMPs)]. For example, in ischemic wounds HBO decreases many MMP levels and increases some tissue inhibitors of MMPs (TIMPs), and so seems to influence proteolytic activity (Zhang and Gould, 2014). Zhang et al. (2008) showed (also in ischemic wounds) that HBO reduced neutrophil infiltration, inflammation, and cell apoptosis. HBO has anti-inflammatory effects in other settings. Sumen et al. (2001) showed that HBO reduced inflammation caused by carrageenan to the same degree as diclofenac. Similar results were found by Wilson et al. (2007) also in a rodent model. In a traumatic brain injury model, Vlodavsky et al. (2006) showed that HBO reduced inflammation in the injured animals treated with HBO compared to controls. Several studies have shown the HBO reduces inflammation and improves outcomes in acute pancreatitis (Christophi et al., 2007; Nikfarjam et al., 2007; Bai et al., 2009). The reactive oxygen and nitrogen species generated by brief HBO exposures may serve as signaling intermediates in several cellular pathways including nitric oxide synthase, HIF-1, VEGF, and heme-oxygenase (HO-1) (Gu et al., 2008). HBO can increase wound growth factor synthesis, mobilize stem cell progenitors from bone marrow, and reduce monocyte chemokine and inflammatory cytokine production (Thom, 2011). Recently, HBO has been shown to be effective in moderately severe ulcerative colitis, another condition in which both inflammation and hypoxia are important contributors to the disease (Dulai et al., 2018). Taken together, the data suggest that HBO is more often beneficial than harmful in acute ischemia, although questions remain.

Another potentially interesting effect of HBO is that it up-regulates anti-oxidant enzymes (superoxide dismutase, catalase, glutathione peroxidase). A growing body of evidence shows that hyperoxia (HBO exposure) prior to hypoxia improves ischemic tolerance. Peng et al. (2008) showed that mice exposed to HBO survived longer in a 10% oxygen environment than mice who had received sham treatments. The pre-treated mice also showed longer swimming times when exposed to a hypoxic environment. Li et al. (2008) showed that antioxidant enzymes were upregulated in rats exposed to hyperoxia, and that they were also able to tolerate ischemia better than controls. Gu et al. (2008) showed upregulation of hypoxia inducible factor-1 alpha and erythropoietin in rats exposed to hyperoxia. These studies also support observations in humans that pretreatment with HBO prior to cardiopulmonary bypass improves outcomes, which may be related to an improved ability to tolerate ischemic stress (Alex et al., 2005; Yogaratnam et al., 2010). These data suggest that HBO might be able to prevent or minimize damage due to acoustic trauma if used prior to exposure. While this may not be a practical approach to preventing acoustic trauma in many settings, research in this area could help reveal the mechanisms underlying cochlear damage with noise or other insults.

## Hydrogen as a Cochlear Therapeutic

Like oxygen, hydrogen also can easily move into the cochlea from the bloodstream. Huang et al. (2010) and Ohta (2014) reviewed the use of hydrogen as a therapeutic gas. Evidence suggests that hydrogen serves as a free radical scavenger and can reduce the strong oxidants hydroxyl radical and peroxynitrite (Kikkawa et al., 2014). Both antioxidant and anti-inflammatory effects have been seen with hydrogen administration in animal models (Huang et al., 2010, 2011). Animals receiving hydrogen in experimentally induced ischemia-reperfusion injury showed less organ damage than controls and studies on the central nervous system, liver, and kidney suggest hydrogen can protect tissue from oxidative stress. Also, hydrogen may affect gene expression, and this may contribute to its anti-apoptotic and anti-inflammatory effects (Ohta, 2014). Overall, these properties suggest that hydrogen may be useful in situations where ROS produce damage.

Since cisplatinum toxicity and acoustic trauma both involve oxidative stress to the cochlea, hydrogen may prove useful in these conditions. Kikkawa et al. (2014) studied cultured mouse cochlear explants exposed to cisplatinum both with and without hydrogen in the culture medium. As expected, cisplatinum caused hair cell loss but hydrogen significantly increased the survival of auditory hair cells. They were also able to show that significantly fewer hydroxyl radicals were produced in the hydrogen-treated cochleae. Fransson et al. (2017) studied the use of hydrogen to protect against cisplatinum induced ototoxicity in a guinea pig model. The guinea pigs breathed hydrogen (2% in air) for 60 min immediately after receiving an injection of cisplatinum. The hydrogen reduced the threshold shifts, prevented inner hair cell loss, and reduced damage to outer hair cells. Chen et al. (2017) examined the use of an abdominal injection of hydrogen-saturated saline on noise-induced hearing loss in a guinea pig model. The guinea pigs that received pre treatment with hydrogen-saturated saline had less hearing loss and the authors concluded this was related to anti-oxidant and anti-inflammatory activity (Chen et al., 2017). Kurioka et al. (2014) had guinea pigs inhale various concentrations of hydrogen for 5 h a day for 5 days after noise exposure. Compared to controls, the hydrogen-treated guinea pigs had better recovery in auditory brainstem response thresholds, less outer hair cell damage, and reduced oxidative DNA damage.

## Other Gases

Carbon dioxide dilates cerebral blood vessels and so perhaps offers a way to increase blood flow to the cochlea after its blood supply has been disrupted after an insult. Carbon dioxide is often given with oxygen (5% carbon dioxide mixed with 95% oxygen). This mixture, called Carbogen, is designed to complement the vasodilating effect of the carbon dioxide with a high level of oxygen to increase oxygen delivery to hypoxic tissues. Dengerink et al. (1984) showed that Carbogen increased cochlear blood flow after it had been reduced by noise exposure. Hatch et al. (1991) studied the relative contributions of carbon dioxide and Carbogen on noise-induced hearing loss in guinea pig model. Carbon dioxide alone (5% carbon dioxide in air) did not provide benefit, while Carbogen and oxygen did, suggesting that the oxygen was more important than the carbon dioxide in reducing noise-induced damage.

Data also suggest that ozone may be useful in preventing or mitigating cochlear damage. Onal et al. (2017) gave ozone for several days to guinea pigs prior to reversible cochlear ischemia, which produced reperfusion injury. The guinea pigs pretreated with ozone had less cochlear damage as well as elevated levels of antioxidant enzymes compared to the control group. The authors concluded that ozone might stimulate endogenous antioxidant defenses leading to these favorable outcomes. Ozone has also been given after a cochlear insult. Kocak et al. (2016) treated rats with intratympanic and rectal ozone for 7 days 1 week after receiving cisplatinum for 3 days to produce ototoxicity. The rats that received ozone showed less outer hair cell and stria vascularis damage and had improved DPOAEs compared to the control rats. The mechanism for this response remains unclear.

Hollig et al. (2014) reviewed the protective effects of argon. Although argon is typically viewed as being inert and non-reactive, a growing body of evidence shows that it can be neuroprotective and organoprotective for tissues exposed to oxygen and glucose deprivation. A human study evaluated argon (24% Ar, 60% N2, 16% O2) in volunteers exposed to 85 dB of white noise for an hour and reported better outcomes with the argon containing mix (Matsnev et al., 2007). The mechanism underlying the neuroprotective and organoprotective effects of argon, however, is not known.

## Conclusion

Although the cochlea has barriers to many molecules and drugs, gases can diffuse readily into the cochlea. If a gas with therapeutic properties can be introduced into the bloodstream it can diffuse into the inner ear and have the desired effect. The two most commonly used gases for treating the cochlear are oxygen and hydrogen, both of which have shown benefit for conditions such as acute acoustic trauma, although many questions remain about the optimal timing, correct dosing, and mechanism of action. Gases such as ozone and argon may also have benefit and deserve further study.

## Author Contributions

JB was responsible for researching and writing the manuscript.

## Conflict of Interest Statement

The author declares that the research was conducted in the absence of any commercial or financial relationships that could be construed as a potential conflict of interest.
